# IL-33 Accelerates Chronic Atrophic Gastritis through AMPK-ULK1 Axis Mediated Autolysosomal Degradation of GKN1

**DOI:** 10.7150/ijbs.93573

**Published:** 2024-04-08

**Authors:** Kewei Liu, Hongxia Huang, Mengyuan Xiong, Qiaojiao Wang, Xiantao Chen, Yinqiong Feng, Hang Ma, Wanqun Chen, Xuegang Li, Xiaoli Ye

**Affiliations:** 1 Engineering Research Center of Coptis Development and Utilization (Ministry of Education), School of Life Sciences, Southwest University, Chongqing, 400715, China.; 2 Daping Hospital, Army Medical University (Third Military Medical University), Chongqing 400042, China.; 3 School of Pharmaceutical Sciences and Chinese Medicine, Southwest University, Chongqing, 400038, China.; 4 Department of Gastroenterology, Chongqing Hospital of Traditional Chinese Medicine, Chongqing, 400000, China.

**Keywords:** IL-33, Autophagy, AMPK, ULK1, GKN1, Chronic Atrophic Gastritis.

## Abstract

Chronic atrophic gastritis (CAG) is a complex disease characterized by atrophy and inflammation in gastric mucosal tissue, especially with high expression of interleukins. However, the interaction and mechanisms between interleukins and gastric mucosal epithelial cells in CAG remain largely elusive. Here, we elucidate that IL-33 stands out as the predominant inflammatory factor in CAG, and its expression is induced by *H. pylori* and MNNG through the ROS-STAT3 signaling pathway. Furthermore, our findings reveal that the IL-33/ST2 axis is intricately involved in the progression of CAG. Utilizing phosphoproteomics mass spectrometry, we demonstrate that IL-33 enhances autophagy in gastric epithelial cells through the phosphorylation of AMPK-ULK1 axis. Notably, inhibiting autophagy alleviates CAG severity, while augmentation of autophagy exacerbates the disease. Additionally, ROS scavenging emerges as a promising strategy to ameliorate CAG by reducing IL-33 expression and inhibiting autophagy. Intriguingly, IL-33 stimulation promotes GKN1 degradation through the autolysosomal pathway. Clinically, the combined measurement of IL-33 and GKN1 in serum shows potential as diagnostic markers. Our findings unveil an IL-33-AMPK-ULK1 regulatory mechanism governing GKN1 protein stability in CAG, presenting potential therapeutic targets for its treatment.

## Introduction

Chronic atrophic gastritis (CAG), a prevailing chronic digestive system disease, is characterized by infiltration of inflammatory cells and atrophy of the gastric mucosa's intrinsic glands, mainly the decrease or disappearance of parietal cells 1-2. According to Correa's cascade process, CAG might proceed to metaplasia and dysplasia, ultimately to gastric cancer, which is one of the most lethal cancers worldwide 3. As patients with CAG are at high risk of progression to gastric cancer, early diagnosis and treatment of CAG is essential for preventing the development of gastric cancer. Although *H. pylori* is considered a primary pathogenic factor for CAG, the etiology of CAG still remains poorly understood.

Chronic inflammation and inflammatory cells play essential roles in a variety of gastrointestinal diseases, including CAG and gastric cancer 4. Specifically, disruptions in the polarization of M1/M2 macrophages induced by *H. pylori* infection may influence the prognosis of CAG 5. And berberine could alleviate CAG by promoting polarization of M2 macrophages while inhibiting M1 macrophages polarization 6. The density of eosinophils is increased in *H. pylori*-induced atrophic gastritis and positively correlated with ulceration and atrophy 7. In addition to immune cells, gastric epithelial cells play critical roles to maintain homeostasis of gastric microenvironment and govern the morbidity of CAG. Specifically, the functions of interleukins in gastric epithelial cells play an important role in the occurrence of CAG. For instance, IL-11 is increased and activates the transcription factor STAT3 to inhibit histamine response by decreasing the expression of ion transporters in epithelial cells during *H. pylori* infection 8. IL-13, released by immune cells, interactes with the receptor on gastric epithelial cells to promote metaplasia 9. IL-17 contributes to metaplasia by inducing apoptosis of parietal cells in CAG 10. Although increasing evidence demonstrate that interleukins participate in the pathogenisis of CAG, the expression profile of interleukins in CAG are still needed to illustrate. And it is worth exploring whether other interleukins can act on gastric epithelial cells to regulate the development of CAG, in order to elucidate the pathogenesis of CAG more clearly.

Autophagy, a highly orchestrated cellular process, serves as a critical mechanism for degradation and recycling of cellular components, including proteins 11. Autophagosomes undergo fusion with lysosomes, resulting in the formation of autolysosomes 12. Subsequently, lysosomal enzymes degrade the encapsulated proteins into their constituent amino acids, which are then recycled by cells 13. The selective removal of proteins through autophagy is essential for cellular maintenance 14. However, dysregulation of autophagy contributes to numerous diseases, including neurodegenerative disease, cancers, infectious and inflammatory disorders 15. While LC3B is increaseed in tissues infected by *H. pylori* 16, the role of autophagy in the development of CAG has not been fully elucidated.

In this study, we show that IL-33 is the most highly expressed interleukin in CAG, and ST2, the receptor of IL-33, is abundantly expressed in gastric epithelial cells, suggesting that IL-33 may act on gastric epithelial cells to participate in the development of CAG. Moreover, the activation of STAT3 triggered by ROS, induced by chemical stimuli or *H. pylori* infection, is the main cause of IL-33 expression. Further studies show that IL-33 promotes autophagy by inducing phosphorylations of AMPK and ULK1 in gastric epithelial cells, thereby accelerating autophagic degradation of GKN1, with the latter playing a pivotal role in inhibiting the development of CAG. Besides, we demonstrate that clearance of ROS to inhibit IL-33 expression or restraining of autophagy may be important potential strategies for the treatment of CAG.

## Materials and Methods

### Cell culture

GES-1 and mouse gastric mucosal epithelial cells were purchased from Procell (Wuhan, China). Cells were maintained in DMEM (Gibco, USA) containing 10% FBS (Gibco, USA), 100 units/ml penicillin and 100 µg/ml streptomycin (Hyclone) in a 5% CO2 atmosphere at 37 °C.

### Reagents and antibodies

The antibodies used in our experiments were: LC3 (CST, #2775), IL-33 (R & D, AF3626 and AF4810), ST2 (Thermofisher, PA5-20077), p-STAT3 (CST, #9145), STAT3 (CST, #9139), p-AMPK (CST, #2535), AMPK (CST, #2532), p-ULK1 (Thermofisher, PA5-105129), ULK1 (Abclonal, A8529), p62 (Abclonal, A1970), GKN-1 (Proteintech, 19344-1-AP), GKN-2 (Abcam, ab188866), GKN-3 (Cloud-clone, PAK528Mu01), Tubulin (Proteintech, 66031-1-Ig).

The chemicals used in our experiments were: Bafilomycin A1 (Selleck, S1413), 3-MA (Selleck, S2767), Rapamycin (Selleck, S1039), MNNG (Selleck, E0157), NAC (Selleck, S1623), Cycloheximide (Selleck, S7418), DAPI (Beyotime, C1002), Recombinant Human IL-33 (R & D, 3625-IL), Pifithrin-α HBr (Selleck, E0157), 2-MeOE2 (Selleck, S1233), ML385 (Selleck, S8790), SR11302 (MCE, HY-15870), BAY 11 (Selleck, S2913), GW9662 (Selleck, S2915), IQ3 (Selleck, S0781), STAT3-IN-7 (Selleck, S0986), FH535 (Selleck, S7484).

### Mouse model and Patients samples

C57BL/6 mice (female, 6 weeks old) were purchased from Gempharmatech China and IL-33 KO mice were kindly gifted from Prof Yuping Lai (Shanghai Key Laboratory of Regulatory Biology, East China Normal University, Shanghai, China). All procedures involving animals were approved by the Animal Ethics Committee of Southwest University and adhered to the Guidelines for Animal Experiments of Laboratory Animals. The ethical approval were obtained on April29, 2022 and the number is IACUC-20220429-10. *H. pylori* (Hp11637) and N-methyl-N'-nitro-N-nitrosoguanidine (MNNG) were employed to establish CAG mouse model. Initially, gastric gavage of *H. pylori* was performed every other day for a total of three times. Following one week of infection, mice were administered drinking water containing 500 μg/ml of MNNG for one week, followed by a week of normal water, and then another week of MNNG water. This cycle was repeated seven times. For mouse models involving the treatement of different inhibitors or inducer, the procedure was followed as described above, with the only variation being that, starting from the first administration of MNNG, mice were orally administered of various inhibitors or inducer (3-MA, 20mg/kg; rapamycin, 20mg/kg; NAC, 20mg/kg) every three days during the induction process. For IL-33 treated mice, the recombinant mouse IL-33 (50μg/kg) was intraperitoneal injected into C57BL/6 mice every three days for eight weeks.

According to the protocols approved by the Institutional Review Board of Chongqing Hospital of Traditional Chinese Medicine and with the informed consent of the patients, the surgically resected fresh tissues were used to detect expressions of IL-33 and GKN1. And serum were used for ELISA measurement of IL-33 and GKN1. The experiment obtained ethical approval from the Chinese Clinical Trial Registry with the approval number ChiCTR1900026455.

### Small interfering RNA (siRNA) and AAV virus

The siRNA targeting *Stat3*, *Ampk*, *Ulk1* were transfected into GES-1 cells by Lipofectmine™ 3000 Transfection Reagent (Invitrogen) according to the manufacturer's instructions. *Stat3* siRNA, 5'-GCAACAGAUUGCCUGCAUUGG-3'; *Ampk* siRNA, 5′-UGCCUACCAUCUCAUAAUATT-3′; *Ulk1* siRNA, 5'-GGAGAAAACUUGUAGGUGU-3'. The AAV-ST2 virus were purchased from Obiosh (Shanghai, China).

### Western blot

Cells were rinsed with PBS and lysed in RIPA buffer supplemented with protease and phosphatase inhibitor cocktails (Bimake, USA). The tissue samples were immersed in RIPA buffer and homogenized using a grinder followed by sonication. Protein concentrations were determined by BCA kit (Beyotime, China) according to the manufacturer's instructions. Total protein were separated by SDS-PAGE followed by transferring onto polyvinylidene difluoride (PVDF) membranes (Millipore, USA). Then PVDF membranes were blocked with 5% skimmed milk for 90 min at room temperature and incubated with indicated primary antibodies at 4 ℃ overnight. After washed by PBST for 3 times, the membranes were probed by HRP-conjugated secondary antibody and visualized by chemiluminescence substrate (Share-Bio, China) with ChemiDoc™ imaging System (Bio-Rad, USA).

### H.E staining

The formalin-fixed, paraffin-embedded tissues were sectioned into 5 micrometer thickness slices. Then the sections were deparaffinized in xylene for 10 minutes, followed by rehydration through a graded ethanol solutions for 3 minutes each. Sebsequently, ections were immersed in hematoxylin for 5 minutes and counterstained with eosin for 2 minutes, followed by rinsing with distilled water. Then slides were observed under a light microscope to obtain the representative images.

### Immunofluorescence staining and confocal microscope

For cyto-immunofluorescence, cells were seeded into Nunc™ Lab-TeK^TM^ Ⅱ Chamber slides. Cells were treated as indicated and then fixed with 4% paraformaldehyde for 20 min at room temperature followed by permeabilization with 0.1% Triton X-100 for 10 min. Cells were incubated with indicated primary antibody at 4 ℃ overnight and fluorochrome-conjugated secondary antibody at room temperature for 1 hour. Fluorescence were observed through the microscope (Zeiss LSM880, Germany).

For tissue immunofluorescence, the formalin-fixed paraffin-embedded samples were deparaffinised and rehydrated through a series of graded ethanol solutions. The slides were boiled in EDTA solution to retrieve antigens and blocked with goat serum. Subsequently, sections were incubated with specified antibodies at 4 ℃ overnight and fluorochrome-conjugated secondary antibody at room temperature for 1 hour. Fluorescence were observed through the microscope (Zeiss LSM880, Germany).

### Phosphoproteomics assay

GES-1 cells were treated by 20 ng/ml IL-33 for 2 hours and lysed with 200 μL lysis buffer (4% SDS, 100 mM DTT, 150 mM Tris-HCl pH 8.0) followed by ultrasonic. After centrifugation at 16,000g for 15 min, the supernatant was collected and quantified by BCA Kit. The proteins were transferred to a 1.5mL Nanosep tube and centrifuged for 20 minutes at 14,000g at 20 ºC. Subsequently, 100 μL of 0.05 M IAA in UA buffer was introduced to block reduced cysteine residues, and samples were incubated for 20 minutes in darkness. Next, 200 µL of Ammonium bicarbonate buffer (100 mM) was added to beads in the filter cartridge. The mixture was centrifuged for 15 minutes at 14,000 x g at 25 ºC. Finally, 50-100 μL of 50 mM Ammonium bicarbonate buffer with trypsin was added to samples, and the mixture was incubated at 37 ℃ for 20 hours. The peptides were collected by centrifugation, acidified with 1% FA, and subsequently dried using a refrigerated CentriVap concentrator (Labconco, Kansas, MO). Ultimately, the tryptic-digested peptides were desalted with a C18 stage tip. Then, phosphopeptides were enriched by High-Select™ TiO2 Phosphopeptide Enrichment kit (Thermo Scientific) and detect by Mass.

### ROS measurement

Cells were seeded into 24-well plates and treated with MNNG or *H. pylori* for 2 hours. Then, cells were exposed to DCFH-DA Superoxide Indicator for 30 min at 37 ℃ in the dark following the manufacturer's introduction. Cells were harvested and prepared for fluorescence measurement by the spectrophotometer via utilizing a 488nm excitation wavelength and a 525nm emission wavelength.

### ELISA assay

The serum of mice were collected and assayed for interleukins, PGA and PGC. Patient's serum were obtained and detected for IL-33 and GKN-1. The production of indicated protein in serum was measured by ELISA kits of mIL-33 (M3300, R & D), hIL-33 (D3300B, R & D), PGA (JL23552, Jonln), PGC (JL23567, Jonln) and GKN-1 (NOV-BG-HUM11042, Neobioscience) according to manufacturer's protocols.

### RNA extraction and RT-qPCR

Total RNA was extracted using Trizol reagent (Invitrogen™) and subjected to reverse transcription using the PrimeScript RT Reagent kit (Takara) to synthesize cDNA following the manufacturer's instructions. Quantitative PCRs were conducted using SYBR premix Ex Taq (Takara) to detect the Ct value of indicated genes on a CFX96 Real Time PCR Detection System (Bio-Rad). The RT-qPCR primers used in these studies are: hIL-33, F-CTACTGCATGAGACTCCGTTCTG, R-AGAATCCCGTGGATAGGCAGAG; mIL-33, F-GCCTGTCAACAGCAGTCTACTG, R-TGTGCTTAGAGAAGCAAGATACTC; mTNF-a, F-GGTGCCTATGTCTCAGCCTCTT, R-GCCATAGA ACTGATGAGAGGGAG; mIL-6, F-TACCACTTCACAAGTCGGAGGC, R-CTGCAAGTGCATCA TCGTTGTTC; mHistamine H2 receptor, F-TCTCTGCTGTCCAGGAGCCAAA, R-TCAGGAG ACAACTTGAGTGACTC; mGKN1, F-CTACCAGAGTGGAGGACCTGAA, R-CCGCAGAATCC AGAGTATGTCAG; mGKN2, F-TCACGAAGGTGGACTGGTTCCT, R-CAGGAGTCCAACCT TTGCACAG; mGKN3, F-GCCTGTGTTCTGGCAAAGATGG, R-GGCTGGGTAAAACTGTGT AGGTC; hATG5, F-GCAGATGGACAGTTGCACACA, R-GAGGTGTTTCCAACATTGGCTCA; hATG7, F-CGTTGCCCACAGCATCATCTTC, R-CACTGAGGTTCACCATCCTTGG; hBeclin1, F-CTGGACACTCAGCTCAACGTCA, R-CTCTAGTGCCAGCTCCTTTAGC.

### Luciferase assay

IL-33 promoter was cloned into pGL3 basic plasmid. Cells were seeded in 24-well plates and transfected with IL-33 promoter luciferase reporter plasmid using lipofectamine3000, along with 10 ng Renilla luciferase reporter plasmid. Cells were treated as indicated and luciferase activity was measured using the Dual Luciferase reporter assay system (Promega). The relative promoter activity was calculated by normalizing firefly luminescence to renilla luminescence.

### Statistical analysis

The immunoblot results and images presented were representative of 3 independent experiments. And GraphPad Prism (version 8) was employed for the statistical analysis of all numeric data. Statistical data were presented as mean ± SEM, with intergroup means analyzed using two-tailed t-test. Multiple comparisons were executed through either one-way ANOVA or two-way ANOVA. The P < 0.05 was considered statistically significant for all statistical tests.

## Results

### IL-33 is the predominant interleukin in chronic atrophy gastritis

**Figure 1:**
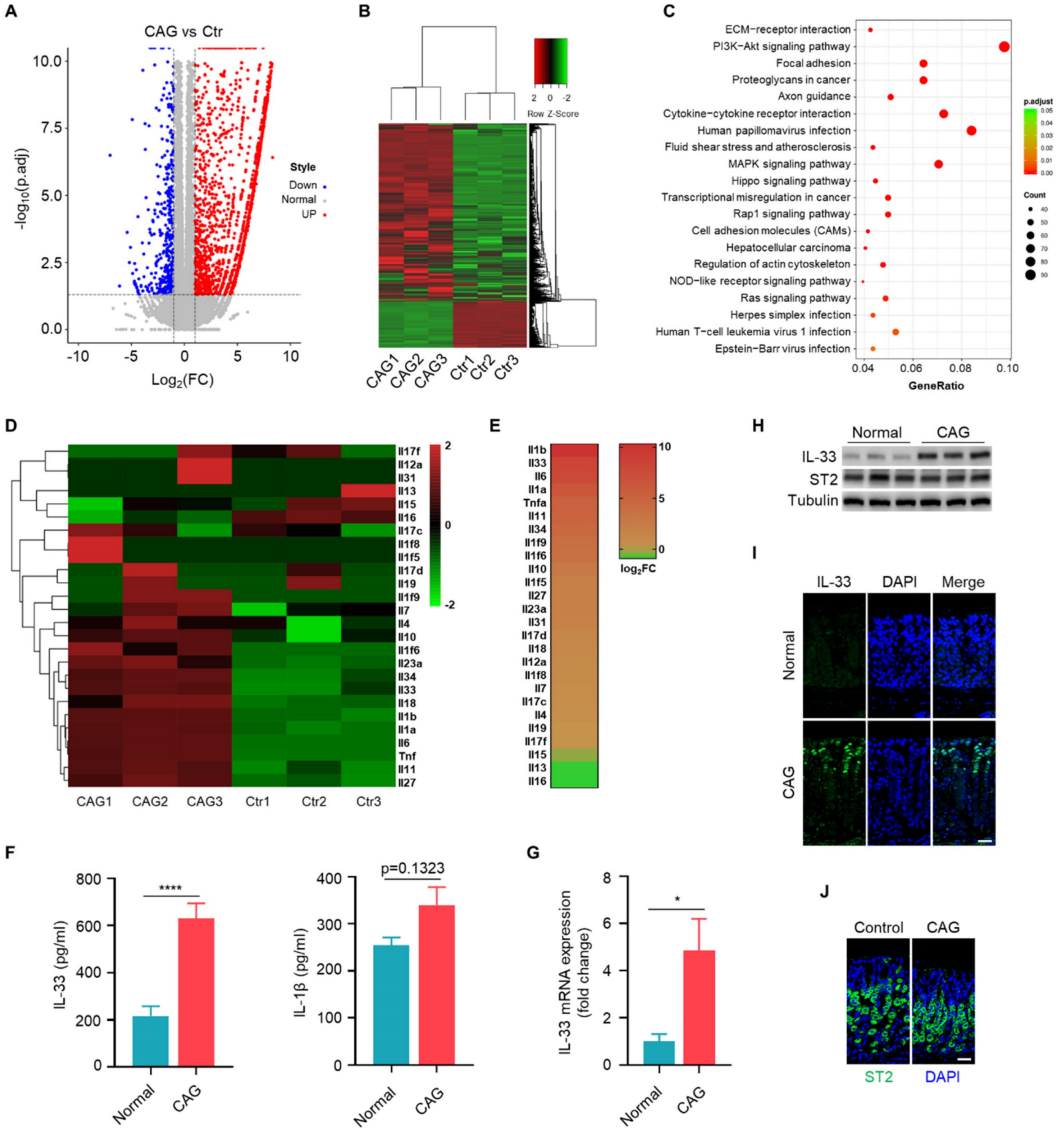
** IL-33 is the predominant interleukin in chronic atrophy gastritis.** (**A**) The volcano plot of differential gene of gastric tissues between CAG mice and Control mice. (**B**) Gene expression clustering heatmap of CAG and normal gastric tissues. (**C**) KEGG pathway analysis based on gene numbers and p-value. (**D**) Differential analysis of the interleukin family in normal and CAG gastric tissues using a heatmap method. (**E**) Expression ranking of interleukins in CAG tissues relative to normal tissues. (**F**) Quantification of IL-33 and IL-1β in serum. (**G**) IL-33 mRNA expression. (H) Western blot of the protein expressions of IL-33 and ST2. (**I**) Immunofluorescence of IL-33 in gastric tissues of normal and CAG mice. Scale bar represents 100μm. (**J**) Immunofluorescence of ST2 in gastric tissues of normal and CAG mice. Scale bar represents 100μm. *p <0.05 and ****p <0.0001. Data are means±s.e.m. and representative of three independent experiments.

We conducted transcriptome sequencing on gastric tissues from CAG mouse model to systematic compare the expression of interleukins in CAG. The scatter of differentially expressed genes (**Fig. 1A**), the cluster analysis (**Fig. 1B**) and KEGG pathway analysis (**Fig. 1C**) based on differentially expressed genes. Subsequently, we performed separate cluster and heatmap analyses for the interleukin family (**Fig. 1D**), ranked the expression of these interleukins and found that IL-1β and IL-33 exhibited the highest fold changes (**Fig. 1E**). However, we observed a relatively weak increase in the protein levels of IL-1β, but a significantly elevation of IL-33 level in the serum of CAG mouse (**Fig. 1**F). Therefore, we aimed to elucidate the mechanism of IL-33 expression and its impact on the pathogenesis of CAG. Furthermore, we validated IL-33 expression in CAG by RT-qPCR and immunoblot (**Fig. 1G and 1H**). Immunofluorescence staining also confirmed elevated IL-33 in CAG, predominantly localized in epithelial mucosal cells (**Fig. 1I**). However, the expression of IL-33 receptor ST2 showed no variation between normal and CAG mice (**Fig. 1H and 1J**). These results collectively demonstrat a significant upregulation of IL-33 expression in CAG.

### *H. pylori* / MNNG induces IL-33 expression via ROS-STAT3 signaling pathway

**Figure 2:**
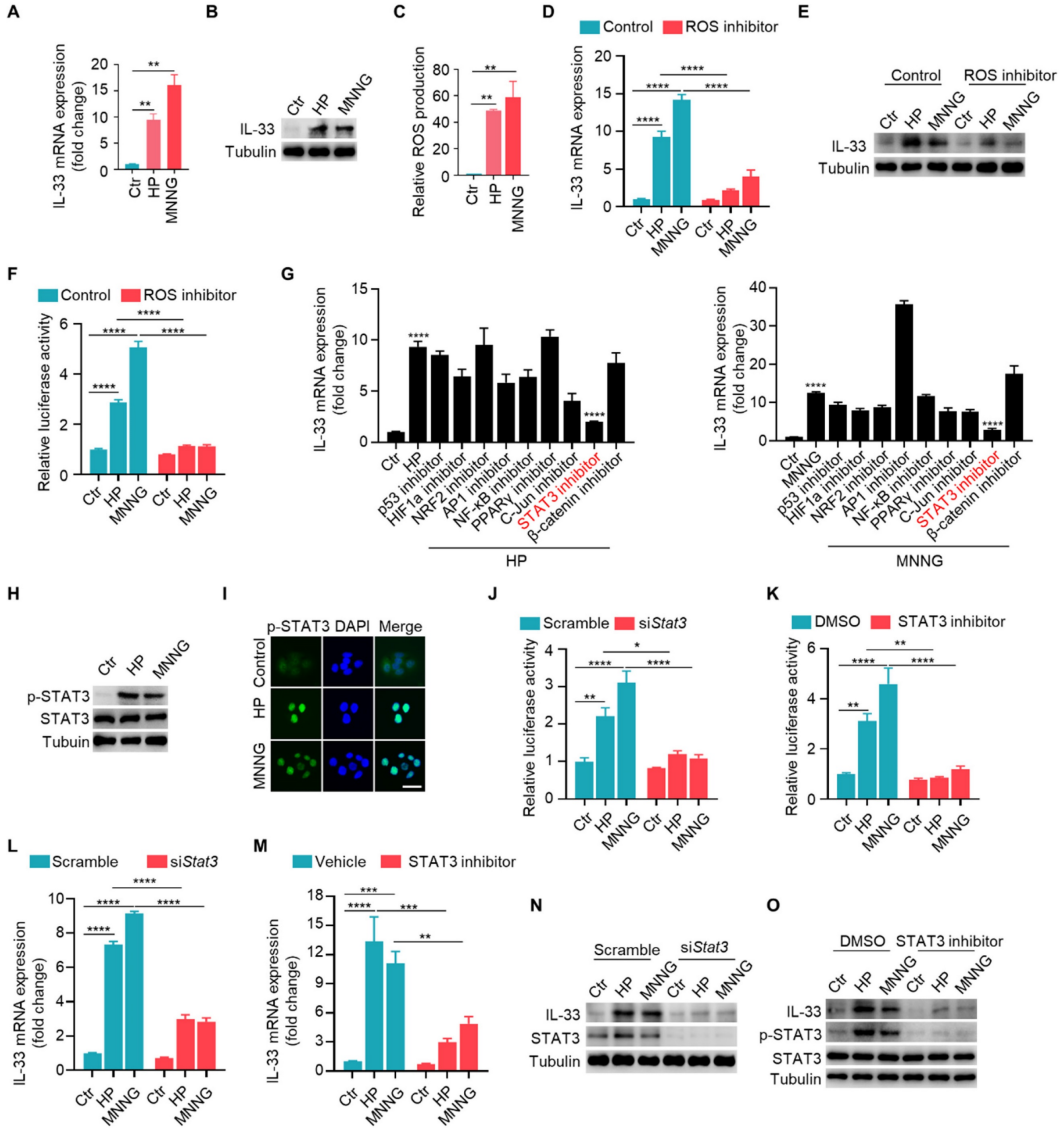
***H. pylori* / MNNG induce IL-33 expression via ROS-STAT3 signaling pathway**. (**A**) IL-33 mRNA expression in GES-1 cells stimulated with 1X10^4^ *H. pylori* or 50 μM MNNG for 12 h. (**B**) IL-33 protein level in GES-1 cells treated as in A for 24 h. (**C**) ROS level in GES-1 cells treated as in A for 3 h. (**D**) IL-33 mRNA expression in GES-1 cells stimulated as in A in the presence or absence of 20μM ROS inhibitor (NAC) for 12 h. (**E**) Protein expression of IL-33 in GES-1 cells treated as in D for 24 h. (**F**) IL-33 promoter activity in GES-1 cells treated as in D. (**G**) IL-33 mRNA expression in GES-1 cells stimulated with 1X10^4^ H.pylori or 50 μM MNNG for 12 h with inhibitors targeting p53 (Pifithrin-α HBr, 10 μM), HIF1α (2-MeOE2, 10 μM), NRF2 (ML385, 10 μM), AP1 (SR11302, 10 μM), NF-κB (BAY 11, 10 μM), PPARγ (GW9662, 10 μM), c-Jun (IQ3, 10 μM), STAT3 (STAT3-IN-7, 10 μM), β-catenin (FH535, 10 μM). (**H**) Immunoblot of p-STAT3 in GES-1 cells stimulated as in A for 6 h. (**I**) Cyto-immunofluorescence of p-STAT3 in GES-1 cells treated as in H for 6 h. Scale bar represents 100μm. (**J**) IL-33 promoter activity in *H. pylori* or MNNG-stimulated GES-1 cells with *Stat3* knockdown by siRNA. (**K**) IL-33 promoter activity in *H. pylori* or MNNG-stimulated GES-1 cells with or without STAT3 inhibitor. (**L**) IL-33 mRNA in GES-1 cells treated as in J for 12 h. (**M**) IL-33 mRNA expression in GES-1 cells treated as in K for 12 h. (**N**) Immunoblot of IL-33 in GES-1 cells treated as in J for 24 h. (**O**) Immunoblot of IL-33 in GES-1 cells treated as in K for 24 h. *p <0.05, **p <0.01 , ***p <0.001 and ****p <0.0001. Data are means±s.e.m. and representative of three independent experiments.

We investigated the mechanisms of elevated IL-33 expression in CAG. *H. pylori* and MNNG were used to construct CAG mouse model. Therefore, we investigated whether *H. pylori* and MNNG could directly induce the expression of IL-33 in gastric epithelial GES-1 cells. We found both *H. pylori* and MNNG induced mRNA and protein expressions of IL-33 in GES-1 cells (**Fig. 2A and 2B**). It have been reported that *H. pylori* infection might be the source of ROS in neutrophils and gastric mucosal cells 17. Additionally, MNNG has been shown to induce ROS production in MEF cells 18. Hence, we quantified the level of ROS in gastric epithelial cells stimulated by *H. pylori* and MNNG, and found that both *H. pylori* and MNNG elicited an increase in ROS production (**Fig. 2C**). ROS elimination by NAC inhibited IL-33 expression induced by *H. pylori* or MNNG (**Fig. 2D and 2E**). And IL-33 promoter activity was dramatically decreased in the presence of NAC (**Fig. 2F**). As ROS can activate a series of transcription factors 19-22, we utilized inhibitors targeting various transcription factors to explore which transcription factor mediated the expression of IL-33 and found that STAT3 was responsible for IL-33 expression (**Fig. 2G**).

Consistently, *H. pylori* and MNNG triggered the phosphorylation and nuclear translocation of STAT3 (**Fig. 2H and 2I**). Besides, we found that when STAT3 was knocked down or inhibited, IL-33 promoter activity was dramatically decreased (**Fig. 2J and 2K**). Furthermore, IL-33 mRNA and protein expressions provoked by *H. pylori* and MNNG were reduced with the depletion or inhibition of STAT3 (**Fig. 2L-2O**). Taken together, these results demonstrate that *H. pylori* and MNNG promote IL-33 expression by activating STAT3 through ROS induction.

### IL-33/ST2 are involved in the pathogenesis of chronic atrophy gastritis

**Figure 3:**
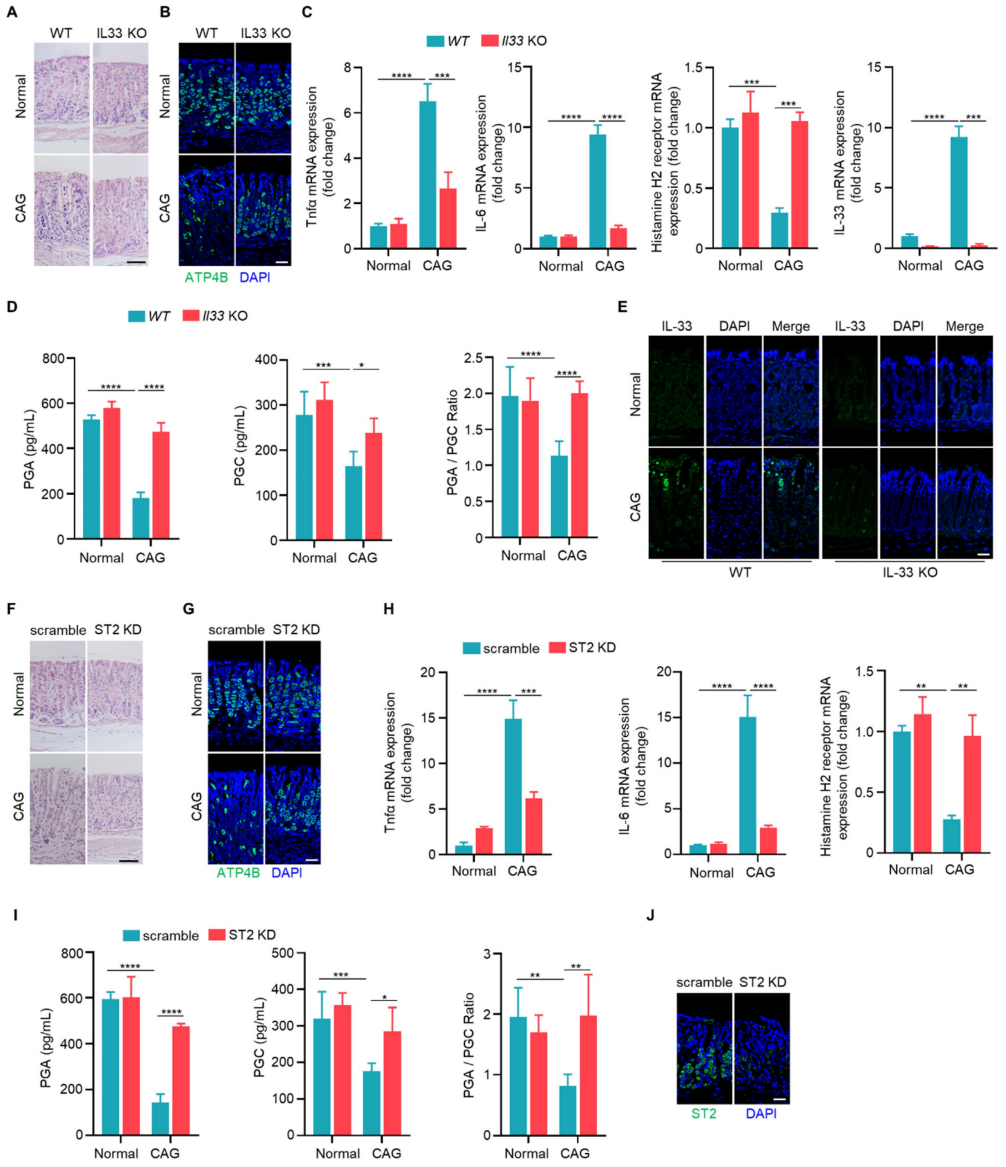
** IL-33/ST2 are involved in the pathogenesis of chronic atrophy gastritis.** (**A**) H.E staining of gastric tissues. Scale bar represents 100 μm. (**B**) Immunofluorescence of ATP4B in gastric tissues. Scale bar represents 100 μm. (**C**) Relative transcript levels of TNF-α, IL-6, histamine H2 receptor and IL-33 in the gastric tissues of WT and IL-33^-/-^ normal or CAG mice. (**D**) Quantification of PGA, PGC, and the ratio of PGA/PGC in serum of WT and IL-33^-/-^ normal or CAG mice. (**E**) immunofluorescence of IL-33. Scale bar represents 100 μm. (**F**) H.E staining of gastric tissues of scramble and ST2 knockdown AAV virus treated normal or CAG mice. Scale bar represents 100 μm. (**G**) Immunofluorescence of ATP4B. Scale bar represents 100 μm. (**H**) mRNA expressions of TNF-α, IL-6, Histamine H2 receptor in the gastric tissues treated as in F. (**I**) Quantification of PGA, PGC, and PGA/PGC ratio in the serum. (**J**) ST2 immunofluorescence staining in gastric tissues. Scale bar represents 100 μm. *p <0.05, **p <0.01 , ***p <0.001 and ****p <0.0001. Data are means±s.e.m. and representative of three independent experiments.

To elucidate the function of IL-33, we induced CAG model in wild-type and IL-33 knockout mice. Upon IL-33 knockout, we observed a reduction in infiltrating inflammatory cells and a lesser loss of parietal cells (**Fig. 3A**). Immunofluorescence staining indicated an increased expression of parietal cell marker ATP4B in CAG model of IL-33 KO mice compared to wild-type mice (**Fig. 3B**). Additionally, the expressions of TNF-α and IL-6 were decreased in IL-33 knockout CAG model (**Fig. 3C**). And the histamine H2 receptor, a low-expressed marker in CAG, was restored after IL-33 knockout (**Fig. 3C**). Furthermore, the levels of pepsinogen A (PGA) and pepsinogen C (PGC), the serum markers decreased expression in CAG, and the ratio of PGA to PGC returned to normal after IL-33 knockout (**Fig. 3D**). The knockout of IL-33 was confirmed through RT-qPCR and immunofluorescence (**Fig. 3C and 3E**). IL-33 is a secretory cytokine that typically exerts its function upon binding to the ligand ST2. Therefore, we further constructed the CAG model after ST2 knockdown by in situ injection of adeno-associated virus targeting ST2. Consistent with the results of IL-33 KO mice, ST2 knockdown led to a reduction in infiltrating inflammatory cells (**Fig. 3F**), an increase in the number of parietal cells (**Fig. 3G**), a decrease in the expressions of TNF-α and IL-6 (**Fig. 3H**), an elevation in histamine H2 receptor expression (**Fig. 3H**), and an increase in serum PGA and PGC levels with normalization of PGA/PGC ratio (**Fig. 3I**). The knockdown efficiency of ST2 was confirmed through immunofluorescence (**Fig. 3J**). Taken together, these findings suggest that IL-33/ST2 promotes the occurrence of CAG.

### IL-33 potentiates autophagy of gastric epithelial cells in an AMPK-ULK1 axis dependent manner

**Figure 4:**
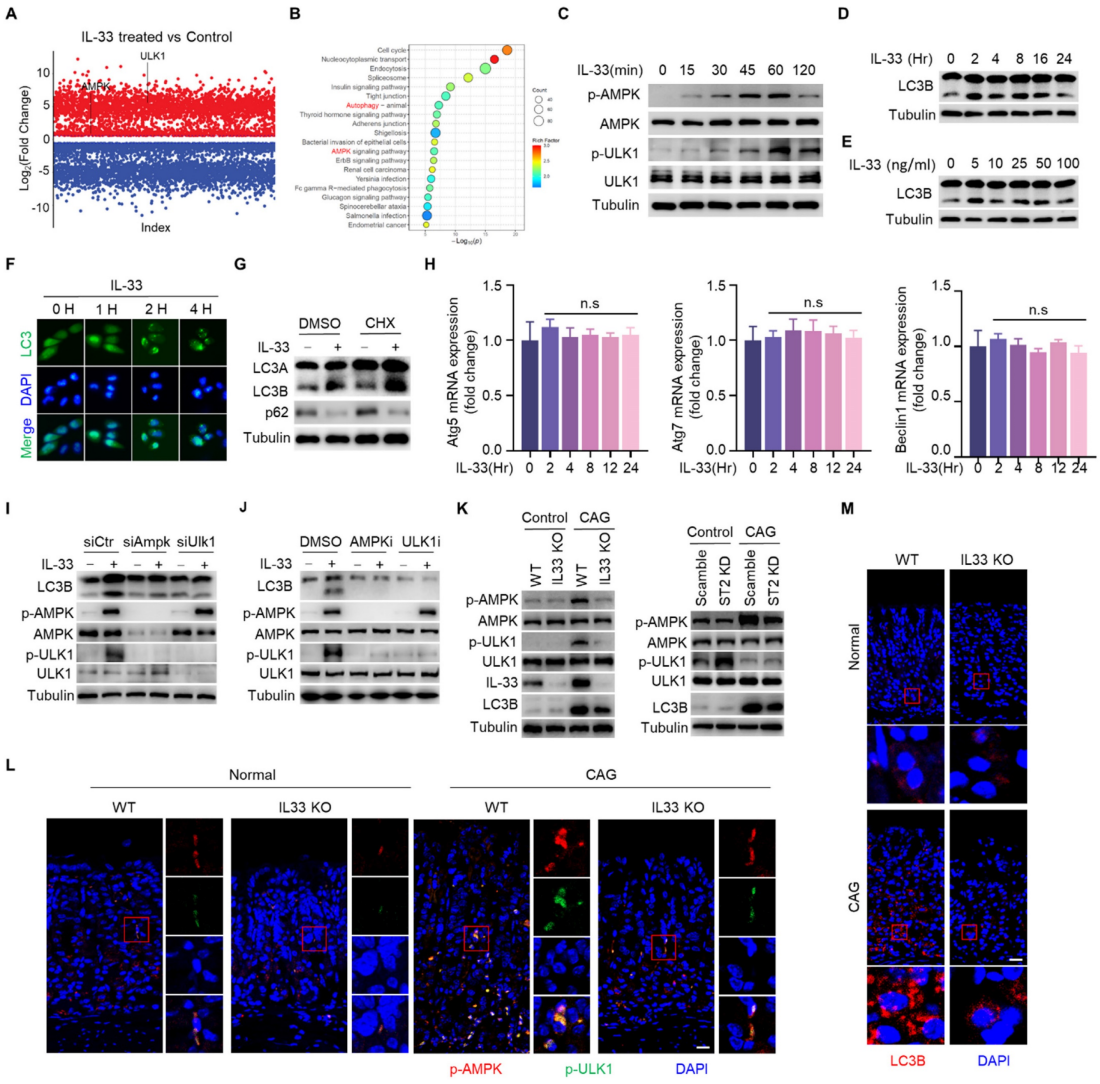
** IL-33 potentiates autophagy of gastric epithelial cells in an AMPK-ULK1 axis dependent manner.** (**A**) Differential phosphorylated proteins obtained from phosphoproteome in GES-1 cells treated by IL-33. (**B**) KEGG enrichment analyse of phosphorylated proteins. (**C**) AMPK and ULK1 phosphorylation in GES-1 cells treated by 20 ng/ml IL-33 for indicated times. (**D**) Immunoblot of LC3 in GES-1 cells treated by 20 ng/ml IL-33 for indicated times. (**E**) Immunoblot of LC3 in GES-1 cells stimulated with different doses of IL-33 for 8 h. (**F**) LC3 puncta in GES-1 cells transfected with GFP-LC3 plasmid for 24 h and treated by 20 ng/ml IL-33 for indicated times. (**G**) Immunoblot of LC3 and p62 in GES-1 cells stimulated with 20 ng/ml IL-33 for 8 h in the presence or absence of 20 μM CHX. (**H**) The mRNA expression of Atg5, Atg7 and Beclin1 in GES-1 cells stimulated with 20 ng/ml IL-33 for indicated times. n.s, no significance. (**I**) Immunoblot of LC3, p-AMPK and p-ULK1 in GES-1 cells transfected with *Ampk* siRNA or *Ulk1* siRNA for 36 h and treated by 40 ng/ml IL-33 for 2 h. (**J**) Immunoblot of LC3, p-AMPK and p-ULK1 in GES-1 cells stimulated by 40 ng/ml IL-33 for 2 h in the presence of 20 μM AMPK inhibitor (AMPK-IN-3) or 20 μM ULK1 inhibitor (ULK-101). (**K**) Immunoblot of p-AMPK, p-ULK1 and LC3 in mice gastric tissues. (**L**) Immunofluorescence of p-AMPK and p-ULK1 in mice gastric tissues. Scale bar represents 100 μm. (**M**) Immunofluorescence of LC3 in mice gastric tissues. Scale bar represents 100 μm.

After binding to its receptor, IL-33 transduces signals into cells by inducing phosphorylation of proteins 23. To investigate the mechanism by which IL-33 promotes the development of CAG, we conducted phosphoproteomic analysis on gastric epithelial cells stimulated by IL-33 for 2 hours. It was found that a large number of phosphorylated proteins changed in IL-33-stimulated cells (**Fig. 4A**), and the phosphorylated proteins were enriched in signaling pathways by KEGG enrichment analyse, including AMPK signaling pathway and autophagy pathway (**Fig. 4B**). Next, we verified that IL-33 could induce phosphorylations of AMPK and ULK1 in a time-dependent manner (**Fig. 4C**). ULK1 is crucial for the initiation of autophagy, and AMPK phosphorylates ULK1 to promote autophagy 24. We further validated that IL-33 could induce autophagy in gastric epithelial cells (**Fig. 4D-4F**). Intriguingly, IL-33 could still induce autophagy in the presence of cycloheximide (CHX) (**Fig. 4G**). Meanwhile, IL-33 had no effect on mRNA expression of autophagy-related genes, including Atg5, Atg7 and Beclin1 (**Fig. 4H**), indicating that IL-33-induced autophagy was independent of new protein synthesis. Furthermore, by silencing AMPK and ULK1 through siRNA, we observed an inhibition of IL-33-induced autophagy (**Fig. 4I**). Additionally, AMPK and ULK1 inhibition resulted in a reduction of IL-33-induced autophagy (**Fig. 4J**). Consistent with those *in vitro* results, *in vivo* findings showed that phosphorylations of AMPK and ULK1, as well as LC3B, were decreased in IL-33 KO or ST2 knock down CAG mice (**Fig. 4K-4M**). These results collectively demonstrate that IL-33 induces autophagy by promoting phosphorylations of AMPK and ULK1.

### Autophagy contributes to the development of chronic atrophy gastritis

**Figure 5:**
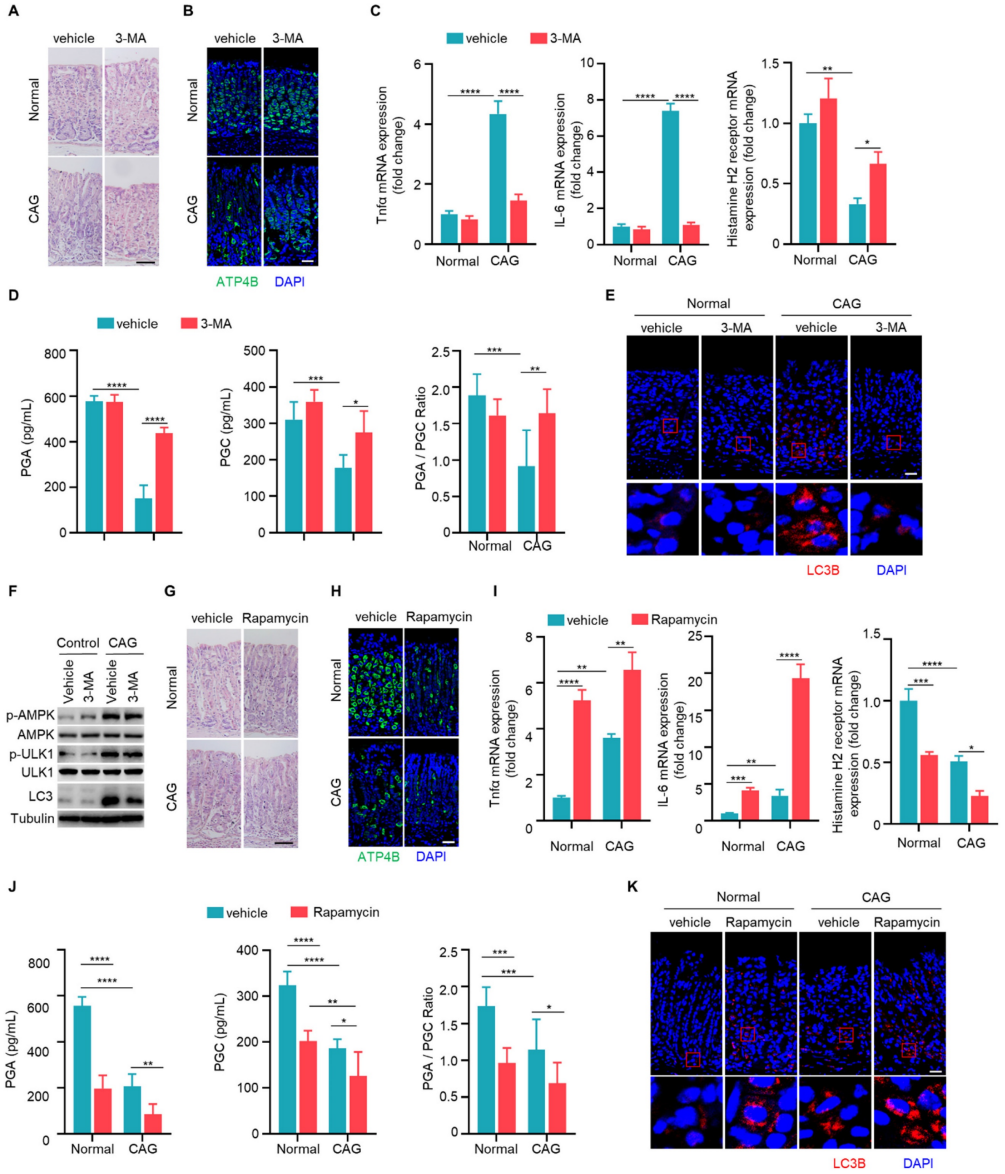
** Autophagy contributes to the development of chronic atrophy gastritis.** (**A**) H.E staining of gastric tissues. Scale bar represents 100 μm. (**B**) Immunofluorescence of ATP4B in gastric tissues. Scale bar represents 100 μm. (**C**) Relative transcript levels of TNF-α, IL-6 and histamine H2 receptor in gastric tissues. (**D**) Levels of PGA, PGC, and the ratio of PGA/PGC in serum. (**E**) Immunofluorescence of LC3 in gastric tissues. Scale bar represents 100 μm. (**F**) Immunoblot of p-AMPK, p-ULK1 and LC3 in gastric tissues. (**G**) H.E staining of gastric tissues. Scale bar represents 100 μm (**H**) Immunofluorescence of ATP4B in gastric tissues. Scale bar represents 100 μm. (**I**) Relative mRNA levels of TNF-α, IL-6 and histamine H2 receptor in gastric tissues. (**J**) Levels of PGA, PGC, and the ratio of PGA/PGC in serum. (**K**) Immunofluorescence of LC3 in gastric tissues. Scale bar represents 100 μm. *p <0.05, **p <0.01, ***p <0.001 and ****p <0.0001. Data are means±s.e.m. and representative of three independent experiments.

Since autophagy is elevated in CAG, we next investigated whether autophagy is involved in the development of CAG. Mice were treated with autophagy inhibitor 3-MA orally every three days during the construction of CAG mouse model. There was a significant reduction in infiltrating inflammatory cells, an increase in the number of parietal cells in CAG group treated with 3-MA (**Fig. 5A and 5B**). Furthermore, we observed that 3-MA dramatically inhibited the expressions of TNF-α and IL-6, reversed the expression of histamine H2 receptor in CAG mice (**Fig. 5C**). Moreover, 3-MA restored PGA and PGC levels in serum of CAG mice, accompanied with normalization of PGA/PGC ratio (**Fig. 5D**). Additionally, 3-MA inhibited autophagy in CAG mice without influencing phosphorylations of AMPK and ULK1 (**Fig. 5E and 5F**).

Subsequently, we assessed whether the autophagy inducer rapamycin could exacerbate severity of CAG. We found that rapamycin alone could lead to the loss of parietal cells. Moreover, in CAG mice subjected to combined treatment with rapamycin, the loss of parietal cells was notably aggravated (**Fig. 5G and 5H**). Additionally, rapamycin alone induced expressions of TNF-α and IL-6 while decreasd expression of histamine H2 receptor (**Fig. 5I**). When used on CAG mice, it augmented the production of pro-inflammatory cytokines and further lead to low expression of histamine H2 receptor (**Fig. 5I**). The serum levels of PGA and PGC exhibited significant reduction, and PGA/PGC ratio was suppressed when rapamycin was administered alone (**Fig. 5J**). Notably, in CAG mice treated with rapamycin, the decrease in both PGA and PGC levels, as well as PGA/PGC ratio, became more pronounced (**Fig. 5J**). Furthermore, when rapamycin is administered in CAG mice, the expression of LC3B increased more significantly compared to CAG mice treated with vehicle or mice treated with rapamycin alone (**Fig. 5K**). All these data indicate that autophagy promotes the occurrence of CAG.

### IL-33 promotes chronic atrophic gastritis through autophagy

**Figure 6:**
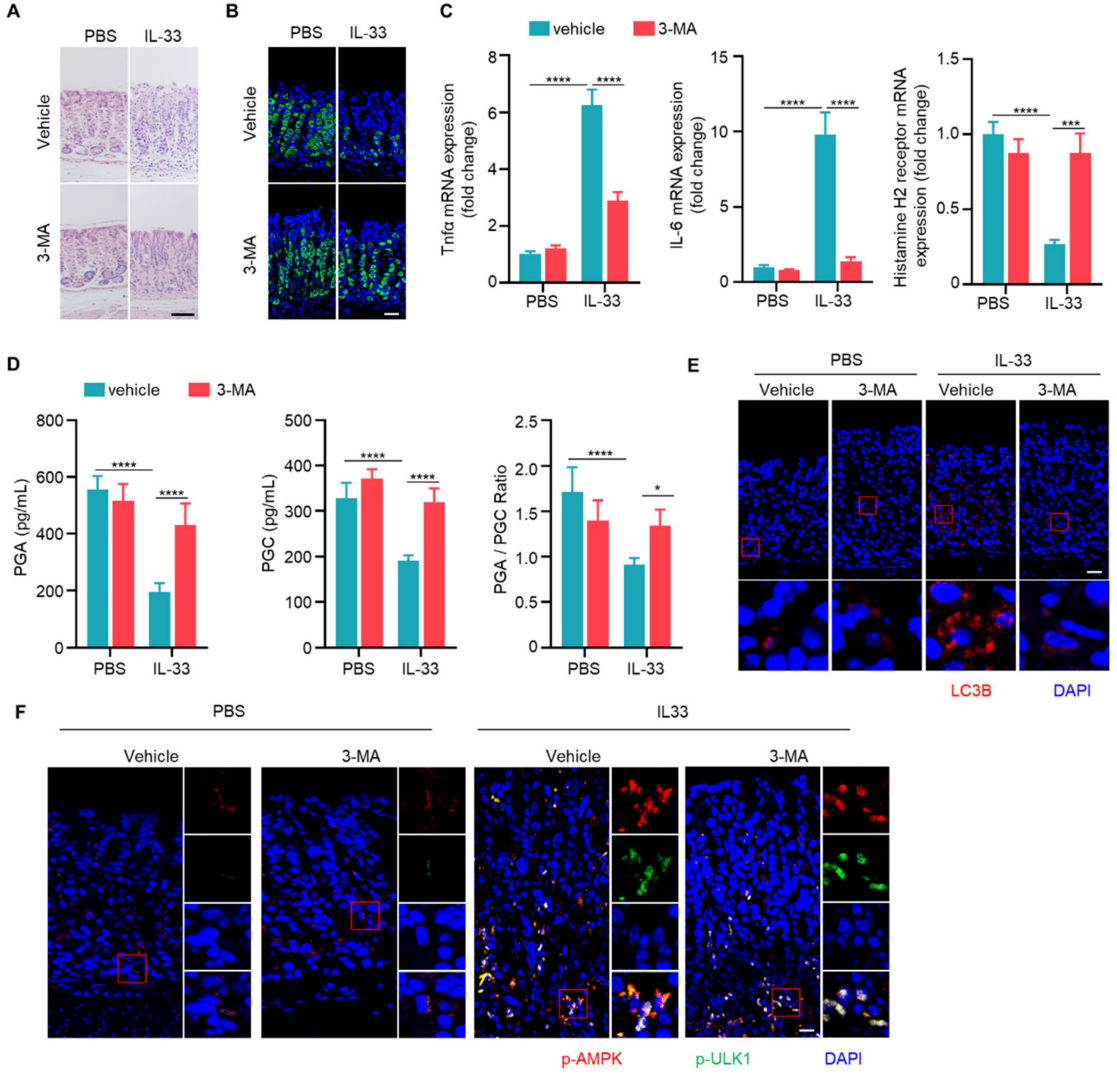
** IL-33 promotes chronic atrophic gastritis through autophagy.** (**A**) H.E staining of gastric tissues of PBS or IL-33 treated mice in the presence or absence of 3-MA. Scale bar represents 100 μm. (**B**) Immunofluorescence of ATP4B in gastric tissues described as in A. Scale bar represents 100 μm. (**C**) Relative transcript levels of TNF-α, IL-6 and histamine H2 receptor in gastric tissues. (**D**) Levels of PGA, PGC, and the ratio of PGA/ PGC in serum. (**E**) Immunofluorescence of LC3 in gastric tissues. Scale bar represents 100 μm. (**F**) Immunofluorescence of p-AMPK and p-ULK1 in gastric tissues. Scale bar represents 100 μm. *p <0.05, ***p <0.001 and ****p <0.0001. Data are means ± s.e.m. and representative of three independent experiments.

Given that both IL-33 and autophagy promote the development of CAG, and IL-33 can induce autophagy, we speculated that IL-33-induced the onset of CAG might depend on autophay. To verify this hypothesis, we injected recombinant IL-33 protein into mice. It was found that IL-33 could lead to infiltration of inflammatory cells and decrease of parietal cells (**Fig. 6A and 6B**). However, simultaneous administration of 3-MA resulted in a reduction of infiltrated inflammatory cells and an increase in the number of parietal cells (**Fig. 6A and 6B**). Furthermore, IL-33 induced expressions of TNF-α and IL-6, inhibited the expression of histamine H2 receptor (**Fig. 6C**). However, the expressions of TNF-α and IL-6 decreased while the expression of histamine H2 receptor increased after autophagy inhibition (**Fig. 6C**).

Importantly, levels of PGA and PGC in serum of IL-33 injected mice decreased, as well as PGA/PGC ratio (**Fig. 6D**). However, co-administration of 3-MA restored levels of PGA, PGC, and the ratio of PGA/PGC (**Fig. 6D**). Besides, IL-33 induced LC3B, while 3-MA inhibited LC3B formation in IL-33 treated mice (**Fig. 6E**). Consistent with *in vitro* results (**Fig. 4C**), IL-33 induced phosphorylations of AMPK and ULK1 *in vivo*, but 3-MA had no effect on phosphorylations of AMPK and ULK1 (**Fig. 6F**). These results collectively suggest that IL-33-induced CAG is dependent on autophagy.

### Scavenging of ROS ameliorates chronic atrophy gastritis by decreasing IL-33 expression and inhibiting autophagy

**Figure 7:**
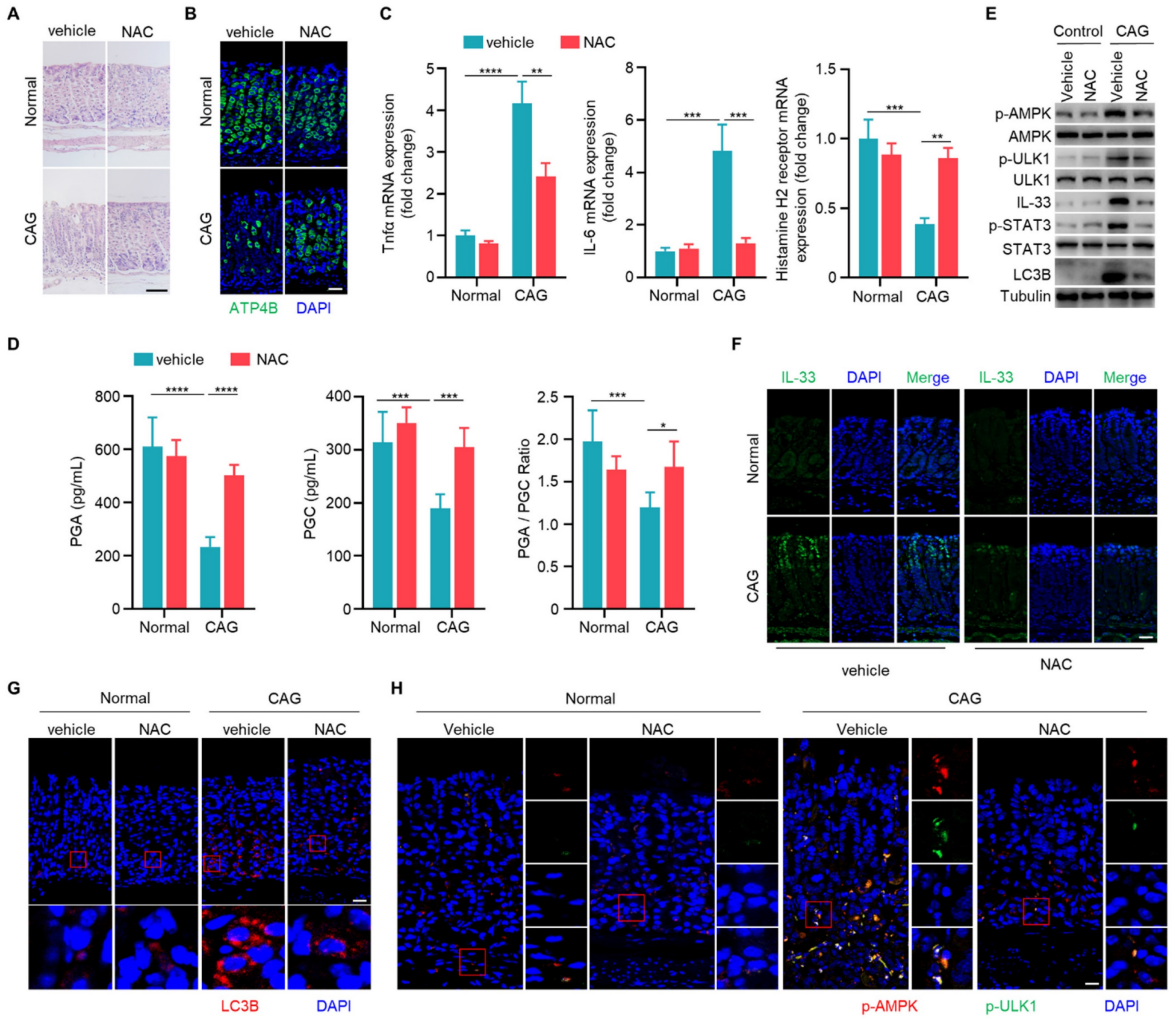
** Scavenging of ROS ameliorates chronic atrophy gastritis by decreasing IL-33 expression and inhibiting autophagy.** (**A**) H.E staining of gastric tissues of normal and CAG mice treated with NAC or vehicle control. Scale bar represents 100 μm. (**B**) Immunofluorescence of ATP4B in gastric tissues. Scale bar represents 100 μm. (**C**) Relative transcript levels of TNF-α, IL-6 and Histamine H2 receptor in the gastric tissues. (**D**) Levels of PGA, PGC, and the ratio of PGA/ PGC in serum. (**E**) Immunoblot of p-AMPK, p-ULK1, p-STAT3, IL-33 and LC3B in gastric tissues. (**F**) Immunofluorescence of IL-33 in gastric tissues. Scale bar represents 100 μm. (**G**) Immunofluorescence of LC3B in gastric tissues. Scale bar represents 100 μm. (**H**) Immunofluorescence of p-AMPK and p-ULK1 in gastric tissues. Scale bar represents 100 μm. *p <0.05, **p <0.01, ***p <0.001 and ****p <0.0001. Data are means±s.e.m. and representative of three independent experiments.

Having established the involvement of IL-33 in the pathogenesis of CAG, and the role of ROS in IL-33 production, we next sought to explore whether scavenging of ROS could alleviate CAG in mouse model. We observed that NAC could increase the number of parietal cells and inhibit infiltration of inflammatory cells in CAG mice (**Fig. 7A and 7B**). Additionally, NAC suppressed expressions of TNF-α and IL-6 while boosted the expression of histamine H2 receptor (**Fig. 7C**). Furthermore, NAC restored levels of PGA, PGC and PGA/PGC ratio in serum of CAG mice (**Fig. 7D**). ROS clearance repressed the activation of STAT3, decreaseed IL-33 expression, suppressed phosphorylations of AMPK and ULK1, and inhibited the formation of LC3B (**Fig. 7E-7H**). These results collectively indicate that clearance of ROS can inhibit the expression of IL-33, thereby suppressing the initiation of autophagy and alleviating CAG.

### IL-33 promotes GKN1 degradation via autolysosomal pathway

**Figure 8:**
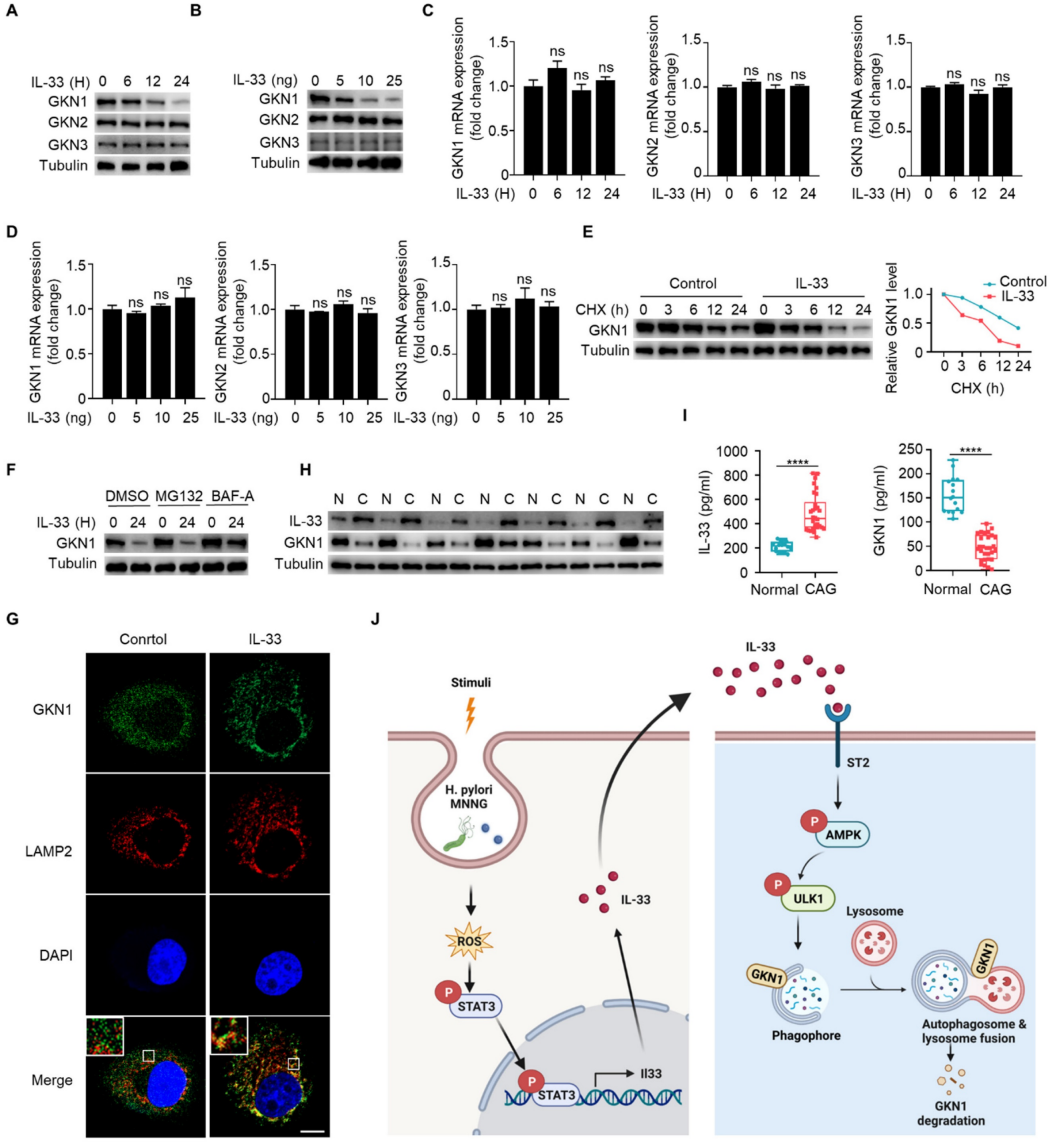
** IL-33 promotes GKN1 degradation via autolysosomal pathway.** (**A**) Immunoblot of GKN1, GKN2 and GKN3 in murine gastric epithelial cells stimulated with 20 ng/ml IL-33 for indicated times. (**B**) Immunoblot of GKN1, GKN2 and GKN3 in murine gastric epithelial cells treated by different doses of IL-33 for 24 h. (**C**) mRNA expression of GKN1, GKN2 and GKN3 in murine gastric epithelial cells stimulated as in A. (**D**) Relative transcript levels of GKN1, GKN2 and GKN3 in murine gastric epithelial cells treated as in B. (**E**) Immunoblot of GKN1 in murine gastric epithelial cells treated by IL-33 and CHX for indicated times (left) and the gray values were quantified (right). (**F**) GKN1 protein expression in murine gastric epithelial cells treated by IL-33 in the presence of MG132 or BAF-A. (**G**) Cytoimmunofluorescence of GKN1 and LAMP2 in murine gastric epithelial cells treated by 20 ng/ml IL-33 for 2 h. Scale bar represents 5 μm. (**H**) Immunoblot of IL-33 and GKN1 in CAG patients. N, non-CAG tissues. C, CAG tissues. (**I**) Quantification of IL-33 and GKN1 production by ELISA in serum from Normal people (n=18) and CAG patients (n=30). (**J**) The schematic model illustrates that high expression of IL-33 induced by ROS-STAT3 axis triggered by stimuli such as *H. pylori* and MNNG in CAG may promote autophagic degradation of GKN1 by phosphorylating AMPK and ULK1. ****p <0.0001. ns, no significance. Data are means±s.e.m. and representative of three independent experiments.

Gastrokine family proteins are highly expressed in epithelial cells of normal gastric mucosa and play crucial roles in gastric diseases, including CAG and gastric cancer 25-26. GKN1 expression is decreased in atrophic gastritis and gastric cancer 27, and GKN3 expression is increased in CAG 28. However, mechanisms governing the alterations of GKNs remain unclear. Since IL-33 can promote development of CAG, whether IL-33 has an effect on the expression of GKN proteins. We found that IL-33 could reduce the protein expression of GKN1 in a time- and dose-dependent manner, while it had no effect on the proteins of GKN2 and GKN3 in gastric epithelial cells (**Fig. 8A and 8B**). In addition, IL-33 had no impact on mRNA expression of GKNs (**Fig. 8C and 8D**). Since IL-33 did not affect GKN1 mRNA expression but reduced its protein level, we speculated that IL-33 might shorten the half-life of GKN1 protein. Indeed, IL-33 significantly decreased the half-life of GKN1 in gastric epithelial cells (**Fig. 8E**). The ubiquitin-proteasome and autophagy-lysosome pathway are main mechanisms for protein degradation 29. And we found that IL-33-promoted degradation of GKN1 was mediated by autolysosome (**Fig. 8F**). Moreover, GKN1 extensively co-localized with the lysosomal marker LAMP2 after IL-33 stimulation (**Fig. 8G**). The above results indicate that IL-33 promotes the degradation of GKN1 through autophagy-lysosome pathway.

Next, we investigated whether IL-33-mediated degradation of GKN1 has clinical significance. We examined protein expressions of IL-33 and GKN1 in fresh tissues from CAG patients, and found that the expression of IL-33 was increased, while the expression of GKN1 was decreased (**Fig. 8H**). Besides, IL-33 level was elevated, while GKN1 level was reduced in serum of CAG patients (**Fig. 8I**). These results suggest that IL-33 and GKN1 might serve as potential markers for the diagnosis of CAG. And this study collectively supports a model that elevated expression of IL-33, induced by *H. pylori* and MNNG through ROS-STAT3, promotes the degradation of GKN1 via AMPK-ULK1 mediated autophagy. It reveals an IL-33-AMPK-ULK1 regulatory mechanism governing GKN1 protein stability in CAG (**Fig. 8J**).

## Discussion

CAG is a complex and multifactorial disease characterized by progressive inflammation and atrophy of gastric mucosa 1. In recent years, there has been a growing interest in understanding the mechanisms underlying the development of CAG 30-31. This study illuminates a novel facet of CAG pathogenesis by examining the involvement of IL-33 and its influence on autophagy through AMPK-ULK1 axis, ultimately leading to autophagic degradation of GKN1.

IL-33, a member of the IL-1 family, is implicated in various inflammatory processes, and its roles in gastric diseases are an emerging area of research 32-33. IL-33 serves as a crucial mediator bridging chronic inflammation and metaplasia through promoting proliferation and recruitment of activated eosinophils in a model predisposed to gastritis 34. IL-33, induced by *H. pylori* in gastric epithelial cell via the ERK pathway, could enhance the inflammatory response of mast cells 35 and recruit eosinophils to produce inflammatory factors 34. However, the role of IL-33 in gastric epithelial cells remains entirely unknown. Here we show that in addition to *H. pylori*, MNNG, a chemical used to induce CAG model, is also capable of inducing IL-33 expression. The common mechanism by which *H. pylori*- and MNNG-induced IL-33 expression involves the production of ROS, subsequently activating STAT3 to enhance IL-33 expression. Additionally, the receptor for IL-33, ST2, is abundantly expressed in various epithelial cell types, such as intestinal epithelial cells 36, respiratory epithelial cells 37, and keratinocytes 38, suggesting that IL-33 might play a role in gastric epithelial cells. Here, we have observed significant expression of ST2 in gastric epithelial cells, indicating the involvement of ST2 in the occurrence of CAG. And IL-33 is mainly expressed by epithelial luminal cells, while ST2 primarily expressed in basal cells of epithelium, thereby forming a paracrine signaling regulatory mechanisms.

The study proposes that IL-33 exerts its effects in gastric epithelial cells through phosphorylating AMPK and ULK1. AMPK, known as a cellular energy sensor, is implicated in the regulation of autophagy, which is a cellular process crucial for maintaining homeostasis through degradation of damaged organelles and proteins 39-40. And activated ULK1 by AMPK is responsible for initiating the formation of autophagosomal 41. IL-33 promotes autophagy and mediates autophagic degradation of STING to protect liver from injury by inducing the expression of Beclin-1 42. IL-33 facilitates autophagy in macrophages to alleviate experimental colitis with the mechanisms underlying IL-33-induced autophagy remaining unknown 43. Our results clarify the role of IL-33 for activation of AMPK and ULK1 to facilitate autophagy in CAG. Although LC3B has increased in tissues of patients with atrophic gastritis infected by *H. pylori* 16, it is not clear whether autophagy promotes the development of CAG. We find that autophagy mediates the promotion effect of IL-33 on CAG.

One of the most intriguing aspects of this study is the autophagic degradation of GKN1 as a consequence of IL-33-induced activation of AMPK-ULK1 axis. GKN1, a secretory protein expressed in gastric epithelial cells, has previously been reported to be associated with gastric mucosal protection and repair 44. Besides, GKN1 has been implicated in the regulation of cellular proliferation and differentiation of gastric epithelium 45. GKN1 is decreased in *H. pylori* infected patients and can be used as a potential biomarker of gastric carcer 46. The expression of GKN1 protein exhibits a progressive downregulation pattern, moving from normal gastric mucosa to atrophic gastritis, intestinal metaplasia, dysplasia, and ultimately to gastric cancer 47. However, the mechanism underlying the downregulation of GKN1 in CAG remains unclear. The observed autophagic degradation of GKN1 suggests a potential loss of its protective functions, providing a mechanistic insight into how IL-33 and autophagy may contribute to the progression of CAG. By simultaneously assessing the levels of IL-33 and GKN1 in serum might serve as potential diagnostic markers for CAG.

The clinical significance of these findings is emphasized through the identification of potential therapeutic targets within the ROS-IL-33-AMPK-ULK1-autophagy axis. Modulating the activities of this axis, including scavenging ROS, suppressing IL-33 expression, and inhibiting autophagy, may provide novel approaches for treating CAG, thereby slowing down or even reversing the progression of gastric mucosal atrophy. It is noteworthy that the study primarily focuses on the autophagic degradation of GKN1 as a downstream consequence of IL-33 signaling. However, autophagy is a multifaceted process with context-dependent outcomes. Further exploration of the broader impact of IL-33-induced autophagy on other cellular components, such as mitochondria and endoplasmic reticulum, could offer a more comprehensive understanding of cellular consequences of IL-33 activation in the gastric mucosa. And conditional knockout mice for ST2 and autophagy genes in gastric epithelial cells are required to further clarify our proposed mechanism. In conclusion, the study significantly contributes to our understanding of CAG pathogenesis by elucidating the role of IL-33 in accelerating gastric mucosal atrophy through AMPK-ULK1 axis-mediated autophagic degradation of GKN1.

## Abbreviations

IL-33: interleukin-33
AMPK: adenosine monophosphate-activated protein kinase
ULK1: unc-51 like autophagy activating kinase 1
ROS: reactive oxygen species
CAG: chronic atrophic gastritis
STAT3: signal transducer and activator of transcription 3
GKN1: gastrokine 1
PGA: pepsinogen A
PGC: pepsinogen C
LC3: microtubule-associated proteins 1A/1B light chain 3
H.pylori: Helicobacter pylori

## References

[1] Song H, Held M, Sandin S, Rautelin H, Eliasson M, Söderberg S (2015). Increase in the Prevalence of Atrophic Gastritis Among Adults Age 35 to 44 Years Old in Northern Sweden Between 1990 and 2009. Clin Gastroenterol Hepatol.

[2] Vannella L, Lahner E, Annibale B (2012). Risk for gastric neoplasias in patients with chronic atrophic gastritis: a critical reappraisal. World J Gastroenterol.

[3] Park YH, Kim N (2015). Review of atrophic gastritis and intestinal metaplasia as a premalignant lesion of gastric cancer. J Cancer Prev.

[4] Wessler S, Krisch LM, Elmer DP, Aberger F (2017). From inflammation to gastric cancer - the importance of Hedgehog/GLI signaling in Helicobacter pylori-induced chronic inflammatory and neoplastic diseases. Cell Commun Signal.

[5] Zhao CN, Xiao LL, Zhang Y (2023). Effects of Helicobacter pylori Infection on the Prognosis of Chronic Atrophic Gastritis by Inducing the Macrophage Polarization. Gastroenterology Res.

[6] Yang T, Wang R, Liu H, Wang L, Li J, Wu S (2021). Berberine regulates macrophage polarization through IL-4-STAT6 signaling pathway in Helicobacter pylori-induced chronic atrophic gastritis. Life Sci.

[7] Aydemir SA, Tekin IO, Numanoglu G, Borazan A, Ustundag Y (2004). Eosinophil infiltration, gastric juice and serum eosinophil cationic protein levels in Helicobacter pylori-associated chronic gastritis and gastric ulcer. Mediators Inflamm.

[8] Howlett M, Chalinor HV, Buzzelli JN, Nguyen N, van Driel IR, Bell KM (2012). IL-11 is a parietal cell cytokine that induces atrophic gastritis. Gut.

[9] Noto CN, Hoft SG, Bockerstett KA, Jackson NM, Ford EL, Vest LS (2022). IL13 Acts Directly on Gastric Epithelial Cells to Promote Metaplasia Development During Chronic Gastritis. Cell Mol Gastroenterol Hepatol.

[10] Bockerstett KA, Osaki LH, Petersen CP, Cai CW, Wong CF, Nguyen TM (2018). Interleukin-17A Promotes Parietal Cell Atrophy by Inducing Apoptosis. Cell Mol Gastroenterol Hepatol.

[11] Glick D, Barth S, Macleod KF (2010). Autophagy: cellular and molecular mechanisms. J Pathol.

[12] Zhao YG, Codogno P, Zhang H (2021). Machinery, regulation and pathophysiological implications of autophagosome maturation. Nat Rev Mol Cell Biol.

[13] Yim WW, Mizushima N (2020). Lysosome biology in autophagy. Cell Discov.

[14] Beese CJ, Brynjólfsdóttir SH, Frankel LB (2020). Selective Autophagy of the Protein Homeostasis Machinery: Ribophagy, Proteaphagy and ER-Phagy. Front Cell Dev Biol.

[15] Klionsky DJ, Petroni G, Amaravadi RK, Baehrecke EH, Ballabio A, Boya P (2021). Autophagy in major human diseases. EMBO J.

[16] Mommersteeg MC, Simovic I, Yu B, van Nieuwenburg SAV, Bruno IMJ, Doukas M (2022). Autophagy mediates ER stress and inflammation in Helicobacter pylori-related gastric cancer. Gut Microbes.

[17] Handa O, Naito Y, Yoshikawa T (2010). Helicobacter pylori: a ROS-inducing bacterial species in the stomach. Inflamm Res.

[18] Chiu LY, Ho FM, Shiah SG, Chang Y, Lin WW (2011). Oxidative stress initiates DNA damager MNNG-induced poly(ADP-ribose)polymerase-1-dependent parthanatos cell death. Biochem Pharmacol.

[19] Ansari RA, Husain K, Rizvi SA (2016). Role of Transcription Factors in Steatohepatitis and Hypertension after Ethanol: The Epicenter of Metabolism. Biomolecules.

[20] Yoon S, Woo SU, Kang JH, Kim K, Kwon MH, Park S (2010). STAT3 transcriptional factor activated by reactive oxygen species induces IL6 in starvation-induced autophagy of cancer cells. Autophagy.

[21] Dharshini LCP, Rasmi RR, Kathirvelan C, Kumar KM, Saradhadevi KM, Sakthivel KM (2023). Regulatory Components of Oxidative Stress and Inflammation and Their Complex Interplay in Carcinogenesis. Appl Biochem Biotechnol.

[22] Chatterjee S, Sil PC (2022). ROS-Influenced Regulatory Cross-Talk With Wnt Signaling Pathway During Perinatal Development. Front Mol Biosci.

[23] Pinto SM, Nirujogi RS, Rojas PL, Patil AH, Manda SS, Subbannayya Y (2015). Quantitative phosphoproteomic analysis of IL-33-mediated signaling. Proteomics.

[24] Egan DF, Shackelford DB, Mihaylova MM, Gelino S, Kohnz RA, Mair W (2011). Phosphorylation of ULK1 (hATG1) by AMP-activated protein kinase connects energy sensing to mitophagy. Science.

[25] Koper-Lenkiewicz OM, Kamińska J, Gawrońska B, Matowicka-Karna J (2019). The role and diagnostic potential of gastrokine 1 in gastric cancer. Cancer Manag Res.

[26] Menheniott TR, O'Connor L, Chionh YT, Däbritz J, Scurr M, Rollo BN (2016). Loss of gastrokine-2 drives premalignant gastric inflammation and tumor progression. J Clin Invest.

[27] Guo XY, Dong L, Qin B, Jiang J, Shi AM (2014). Decreased expression of gastrokine 1 in gastric mucosa of gastric cancer patients. World J Gastroenterol.

[28] Bockerstett KA, Lewis SA, Noto CN, Ford EL, Saenz JB, Jackson NM (2020). Single-Cell Transcriptional Analyses Identify Lineage-Specific Epithelial Responses to Inflammation and Metaplastic Development in the Gastric Corpus. Gastroenterology.

[29] Varshavsky A (2017). The Ubiquitin System, Autophagy, and Regulated Protein Degradation. Annu Rev Biochem.

[30] Zheng SY, Zhu L, Wu LY, Liu HR, Ma XP, Li Q (2023). Helicobacter pylori-positive chronic atrophic gastritis and cellular senescence. Helicobacter.

[31] Liu H, Li PW, Yang WQ, Mi H, Pan JL, Huang YC (2019). Identification of non-invasive biomarkers for chronic atrophic gastritis from serum exosomal microRNAs. BMC Cancer.

[32] Lv Y, Tian W, Teng Y, Wang P, Zhao Y, Li Z (2023). Tumor-infiltrating mast cells stimulate ICOS+ regulatory T cells through an IL-33 and IL-2 axis to promote gastric cancer progression. J Adv Res.

[33] Eissmann MF, Dijkstra C, Jarnicki A, Phesse T, Brunnberg J, Poh AR (2019). IL-33-mediated mast cell activation promotes gastric cancer through macrophage mobilization. Nat Commun.

[34] De Salvo C, Pastorelli L, Petersen CP, Buttò LF, Buela KA, Omenetti S (2021). Interleukin 33 Triggers Early Eosinophil-Dependent Events Leading to Metaplasia in a Chronic Model of Gastritis-Prone Mice. Gastroenterology.

[35] Lv YP, Teng YS, Mao FY, Peng LS, Zhang JY, Cheng P (2018). Helicobacter pylori-induced IL-33 modulates mast cell responses, benefits bacterial growth, and contributes to gastritis. Cell Death Dis.

[36] He Z, Chen L, Furtado GC, Lira SA (2018). Interleukin 33 regulates gene expression in intestinal epithelial cells independently of its nuclear localization. Cytokine.

[37] Li YQ, Zhong Y, Xiao XP, Li DD, Zhou Z, Tian YY (2020). IL-33/ST2 axis promotes the inflammatory response of nasal mucosal epithelial cells through inducing the ERK1/2 pathway. Innate Immun.

[38] Wu Y, Quan Y, Liu Y, Liu K, Li H, Jiang Z (2016). Hyperglycaemia inhibits REG3A expression to exacerbate TLR3-mediated skin inflammation in diabetes. Nat Commun.

[39] Li J, Zhong L, Wang F, Zhu H (2017). Dissecting the role of AMP-activated protein kinase in human diseases. Acta Pharm Sin B.

[40] Guo R, Ren J (2012). Deficiency in AMPK attenuates ethanol-induced cardiac contractile dysfunction through inhibition of autophagosome formation. Cardiovasc Res.

[41] Egan DF, Shackelford DB, Mihaylova MM, Gelino S, Kohnz RA, Mair W (2011). Phosphorylation of ULK1 (hATG1) by AMP-activated protein kinase connects energy sensing to mitophagy. Science.

[42] Wang Z, Sun P, Pan B, Qiu J, Zhang X, Shen S (2023). IL-33/ST2 antagonizes STING signal transduction via autophagy in response to acetaminophen-mediated toxicological immunity. Cell Commun Signal.

[43] Wang Z, Shi L, Hua S, Qi C, Fang M (2019). IL-33 ameliorates experimental colitis involving regulation of autophagy of macrophages in mice. Cell Biosci.

[44] Kim O, Yoon JH, Choi WS, Ashktorab H, Smoot DT, Nam SW (2014). GKN2 contributes to the homeostasis of gastric mucosa by inhibiting GKN1 activity. J Cell Physiol.

[45] Yan GR, Xu SH, Tan ZL, Yin XF, He QY (2011). Proteomics characterization of gastrokine 1-induced growth inhibition of gastric cancer cells. Proteomics.

[46] Nardone G, Martin G, Rocco A, Rippa E, La Monica G, Caruso F (2008). Molecular expression of Gastrokine 1 in normal mucosa and in Helicobacter pylori-related preneoplastic and neoplastic gastric lesions. Cancer Biol Ther.

[47] Yoon JH, Ham IH, Kim O, Ashktorab H, Smoot DT, Nam SW (2018). Gastrokine 1 protein is a potential theragnostic target for gastric cancer. Gastric Cancer.

